# Supplementary material for: Edge phonons in black phosphorus
Source: Nat Commun. 2016 Jul 14;7:12191. doi: 10.1038/ncomms12191 (PMC4947165; doi:10.1038/ncomms12191)
Supplement: Supplementary Information — Supplementary Figures 1-13, Supplementary Table 1, Supplementary Note 1-2 and Supplementary References [file ncomms12191-s1.pdf]

SUPPLEMENTARY INFORMATION

SUPPLEMENTARY FIGURES

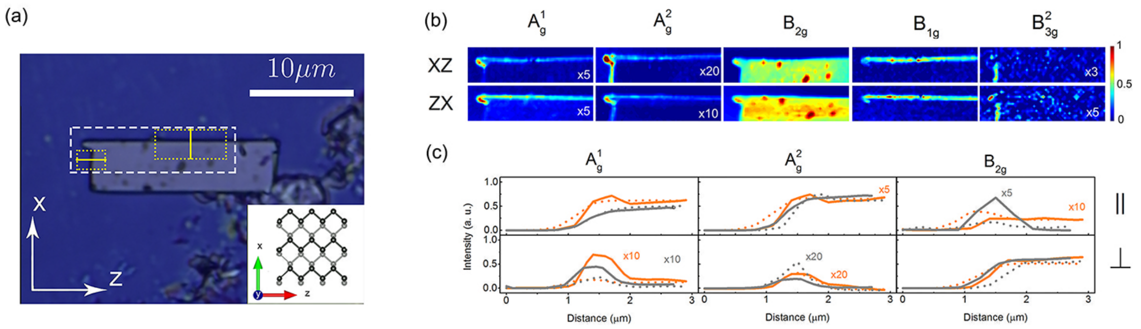

Supplementary Figure 1. Optical and hyperspectral Raman images of an exfoliated black phosphorus flake with armchair and zigzag edges. (a) Optical microscope image of the measured flake; the white dashed rectangle defines the analysed area. The corresponding crystal orientation and crystallographic axes are presented in the inset. (b) Hyperspectral Raman intensity images of all observed modes (columns), for the XZ and ZX configurations (indicated on the left). Mode intensities are represented by the colorbar and were normalized to the highest value of each mode. Whenever necessary, for better visualization, the intensity was multiplied by a factor, as indicated at the bottom-right corner of each image. (c) Intensity profiles along the yellow lines shown in (a) for the A_g^1, A_g^2 and B_{2g} modes. Columns indicate the different analysed modes and rows correspond to the parallel and orthogonal incident/analysed polarisation configurations. Orange and grey curves indicate z and x incident polarisation, respectively. Solid and dashed curves are the profiles taken at the zigzag and armchair edges, respectively.

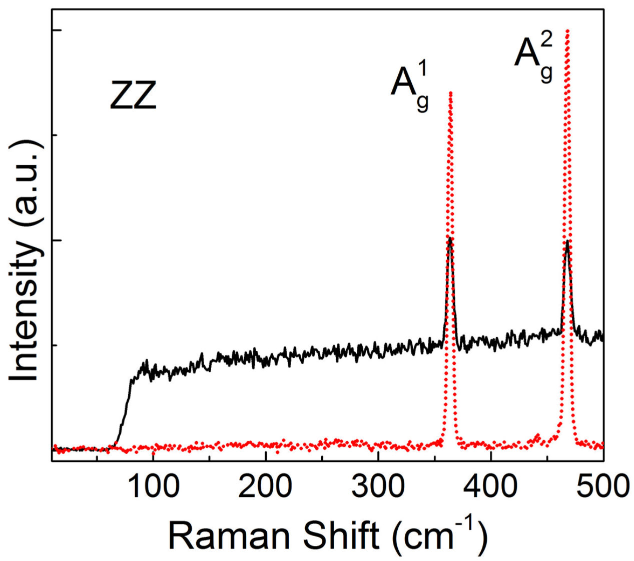

Supplementary Figure 2. **Raman spectrum at and away from a defective spot.** Raman spectrum obtained with the ZZ configuration at one of the bright spots seen in Fig. 1(d) (solid black line) and spectrum of a perfect region within the interior of the sample (dotted red line).

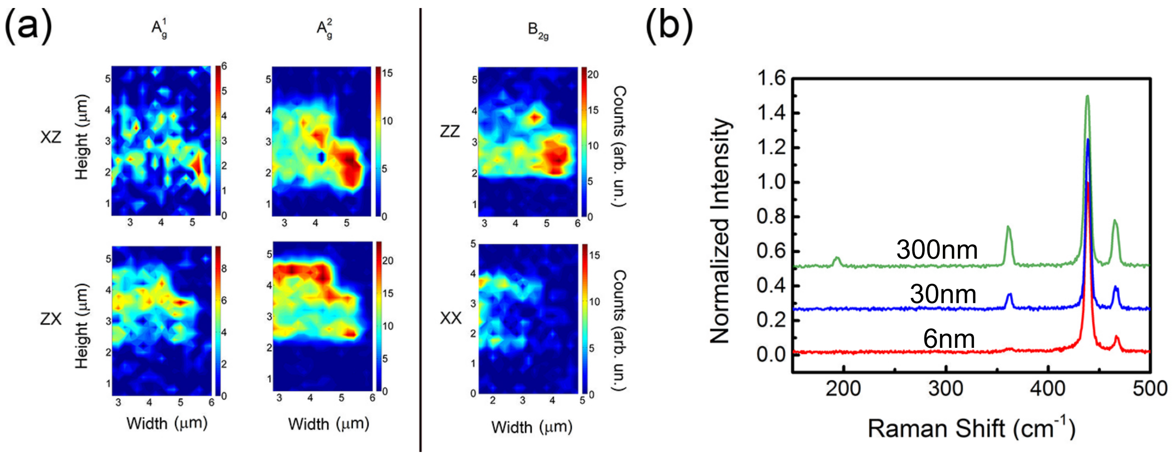

Supplementary Figure 3. **Hyperspectral Raman images for a 6-nm-thick flake and Raman spectra for flakes with different thicknesses.** (a) Hyperspectral Raman images for a 6-nm thick flake. Columns correspond to modes A_g^1, A_g^2 and B_{2g}, while lines correspond to the XZ and ZX or to the ZZ and XX configurations, according to the labels. (b) Raman spectra for three flake thicknesses at an armchair edge for the ZX configuration. The spectra were normalized by the B_{2g} mode intensity and were vertically shifted for better visualization. From bottom to top, the flake thicknesses are 6, 30 and 300 nm.

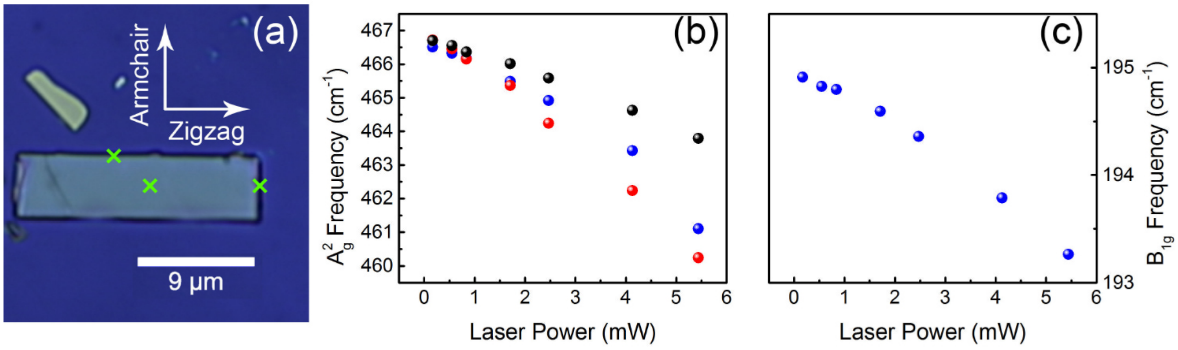

Supplementary Figure 4. **Raman shift laser power (temperature) dependence at black phosphorus edges and at the flake centre.** (a) Optical image of a rectangular flake in which Raman measurements were made as functions of laser power (ZZ configuration used). (b) A_g^2 mode spectral positions as functions of the laser power at the center (black), armchair (blue), and zigzag (red) positions marked with crosses in (a). (c) B_{1g} mode spectral position as a function of laser power at the armchair edge.

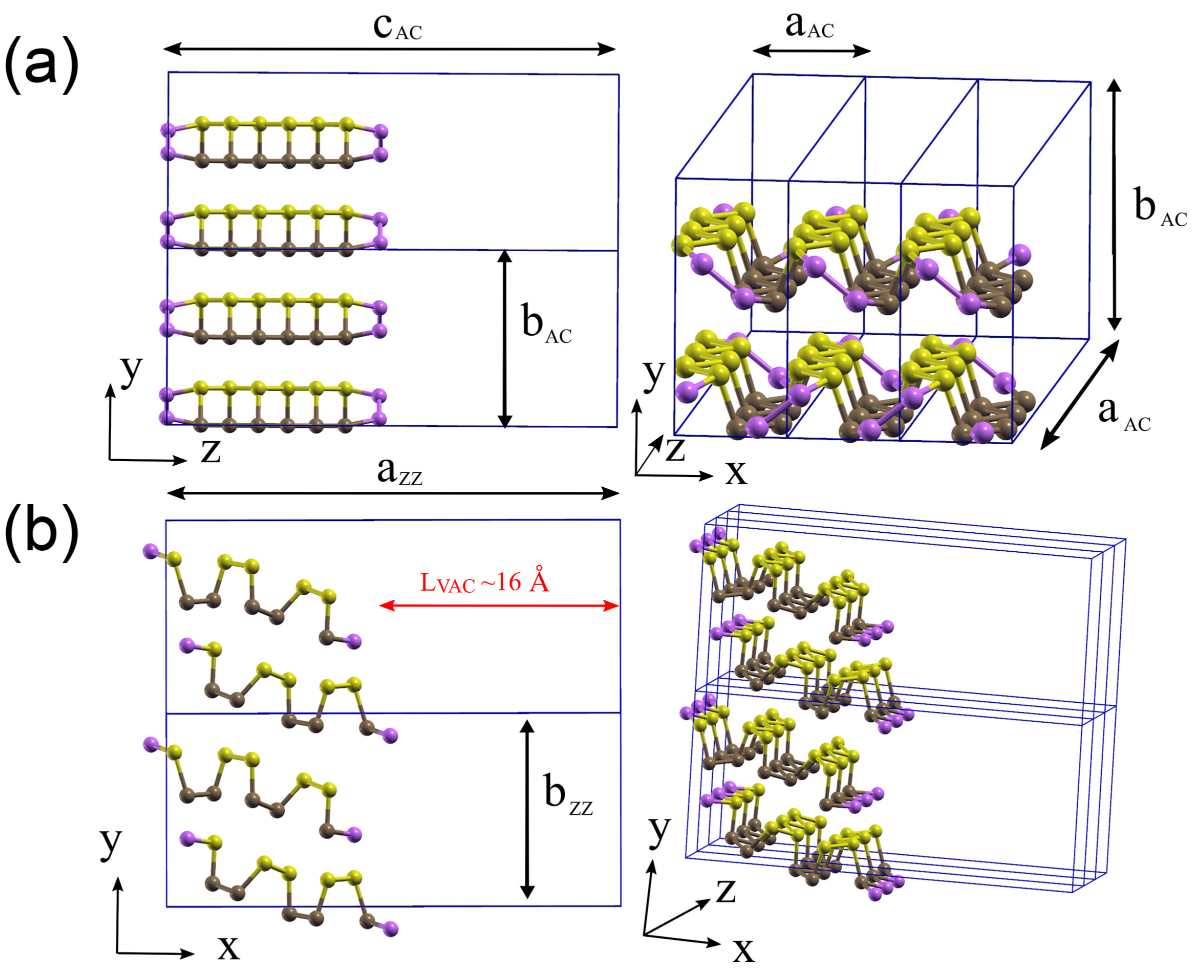

Supplementary Figure 5. **Various views of relaxed simulated slab structures.** Structure obtained after relaxation with different perspectives highlighting the unit cell for (a) armchair, and (b) zigzag structures.

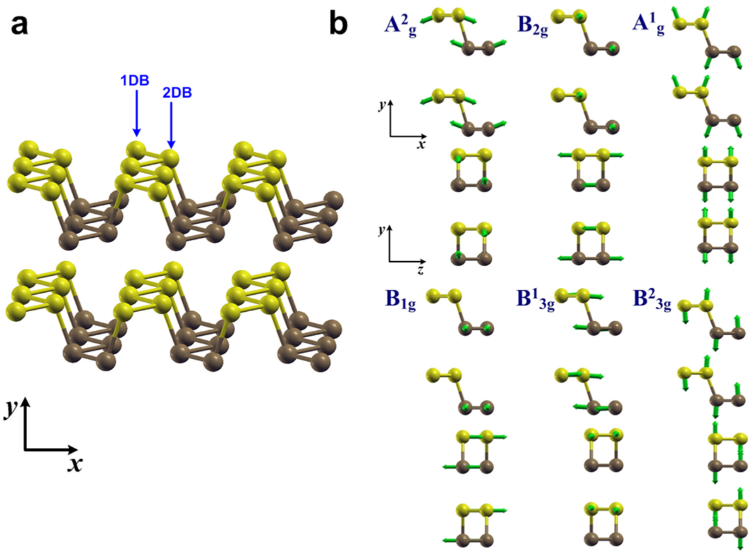

Supplementary Figure 6. Black phosphorus structure and Raman mode atomic displacements. (a) Schematic representation of black phosphorus showing its puckered structure. Blue arrows indicate the possible atom termination of a zigzag surface. (b) Side views of the atomic displacements for the Raman active modes. The green arrows represent the amplitude of the displacements.

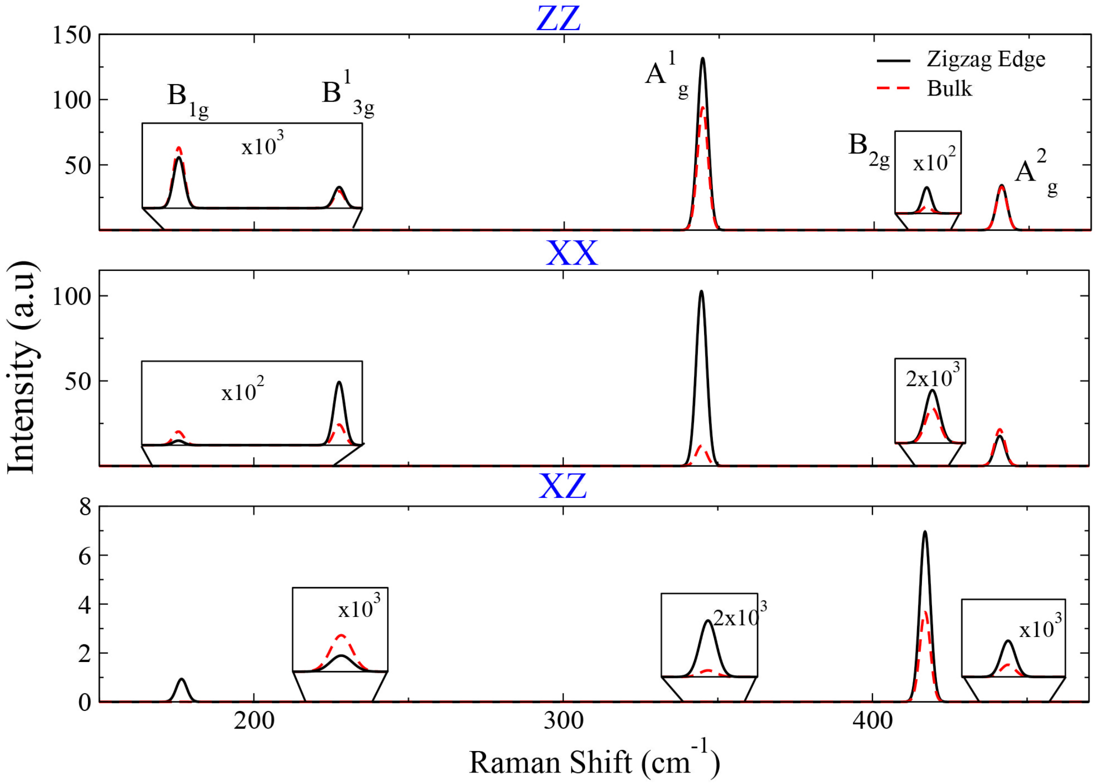

Supplementary Figure 7. **Square root of the calculated Raman intensities for a zigzag slab.** From top to bottom: ZZ, XX, and ZX scattering configurations, respectively. The solid black lines indicate the spectra for the zigzag edge and the red dashed lines stand for the spectra for the bulk.

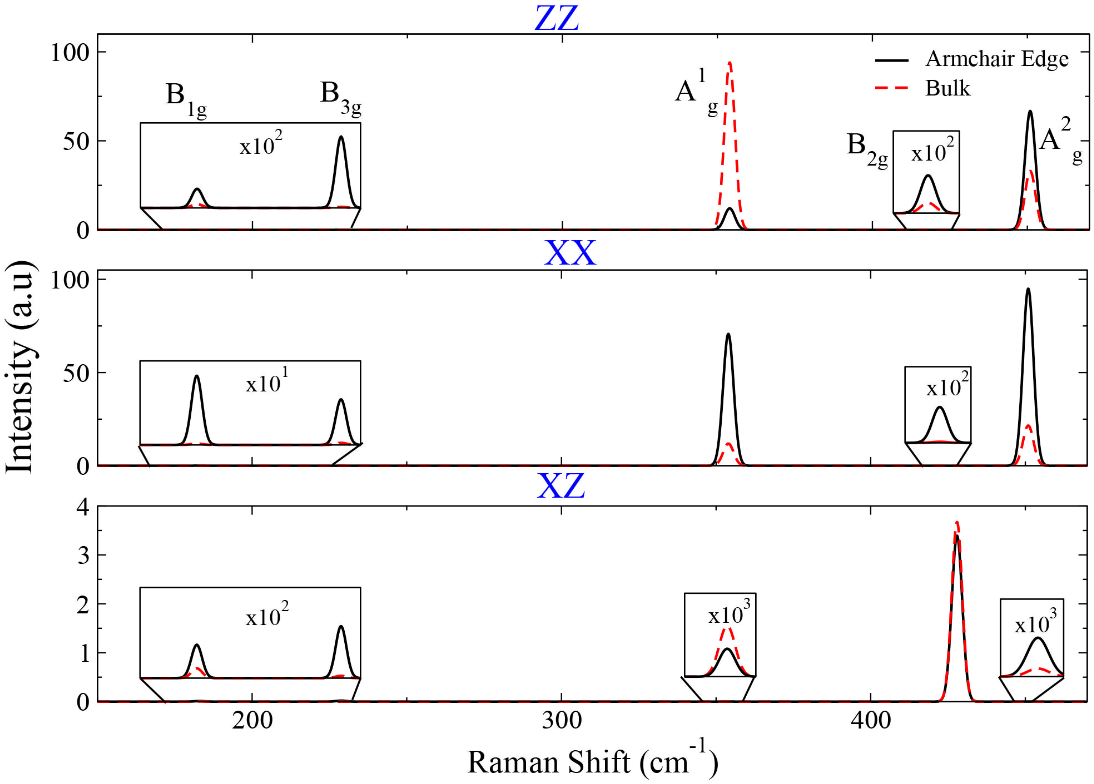

Supplementary Figure 8. **Square root of the calculated Raman intensities for an armchair slab.** From top to bottom: ZZ, XX, and ZX scattering configurations, respectively. The solid black lines indicate the spectra for the armchair edge and the red dashed lines stand for the spectra for the bulk.

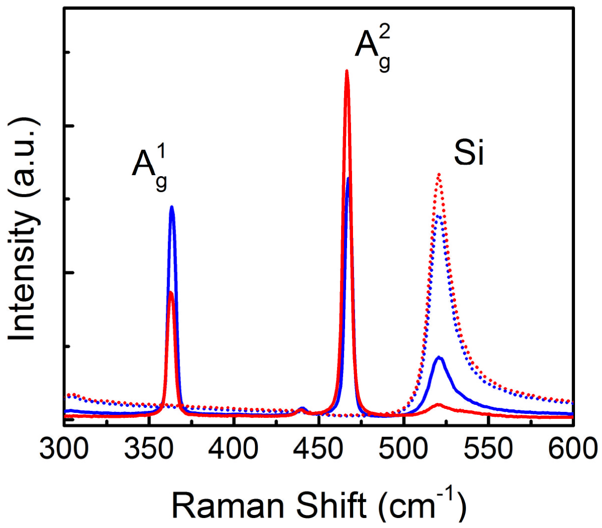

Supplementary Figure 9. Polarized Raman spectra of black phosphorus and of the silicon substrate, used for flake orientation. Raman spectra obtained through (solid) and away from (dashed) a 170-nm thick BP flake with polarization parallel to BPs two crystallographic axes (red and blue). From the lower Si band amplitude measured through BP, the polarization used for obtaining the red curves is found to be parallel to the armchair direction.

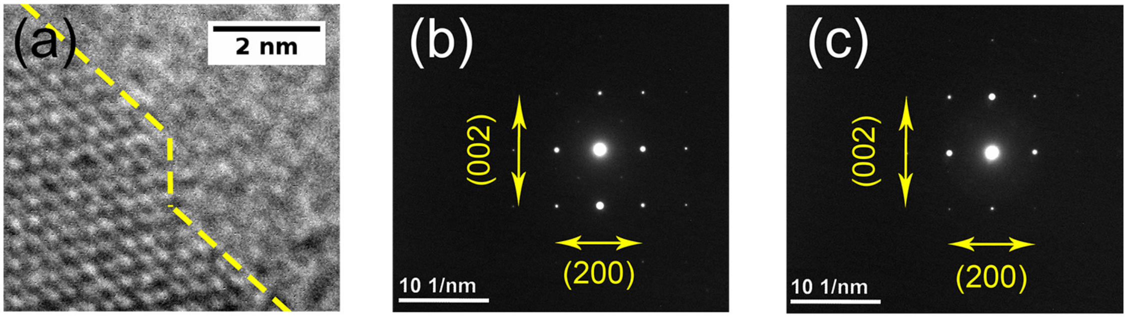

Supplementary Figure 10. Characterization of a black phosphorus flake via transmission electron microscopy and diffraction. (a) HRTEM image; electron diffraction patterns obtained at the edge (b) and in the interior of the flake (c).

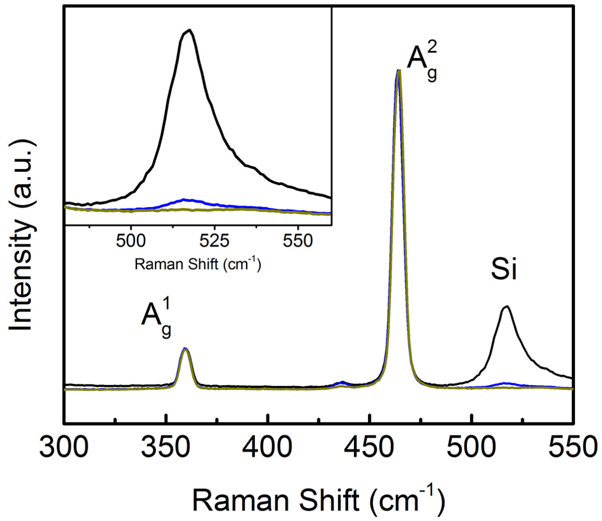

Supplementary Figure 11. **Raman spectra of black phosphorus flakes with different thicknesses.** 16 nm (green), 65 nm (blue), and 117 nm (black), all in the XX configuration.

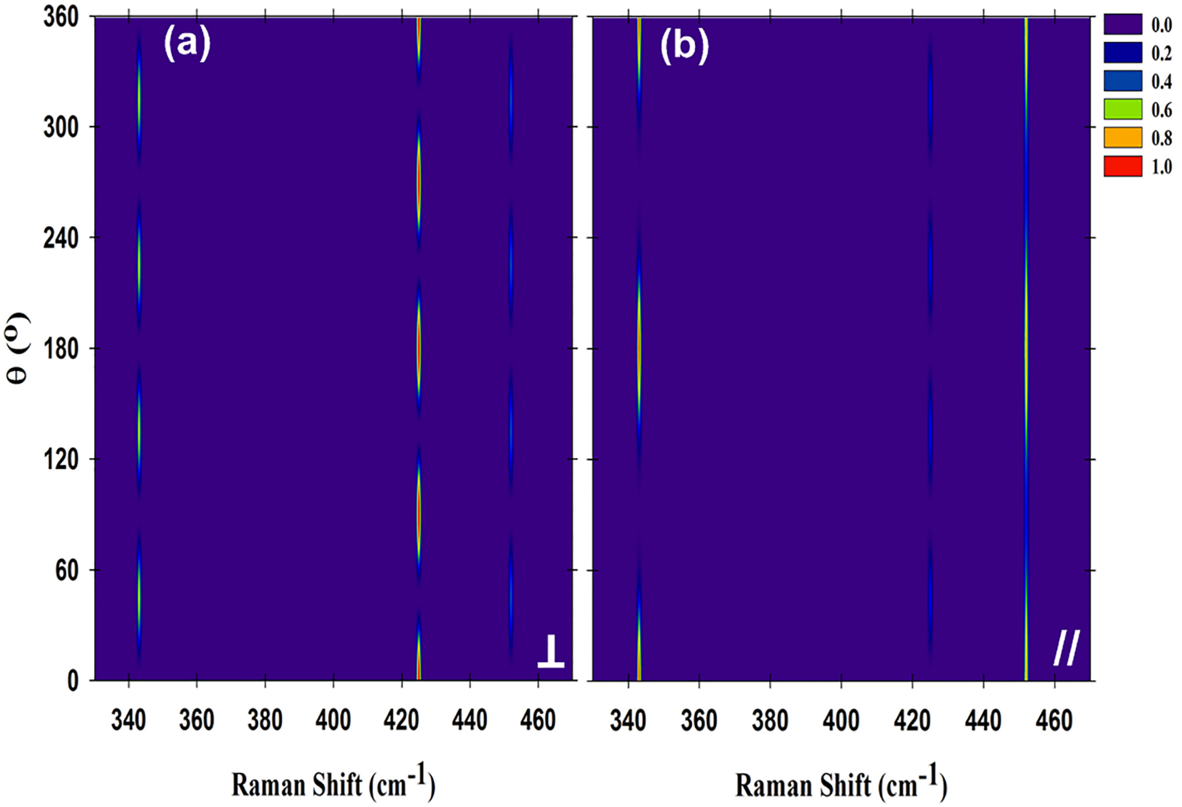

Supplementary Figure 12. **Calculated angular dependence of the polarized Raman spectrum in bulk black phosphorus.** Raman spectra as functions of the angle of incident polarisation (with respect to the z-axis) for scattered polarisations (a) perpendicular, and (b) parallel to the incident polarisation. The Raman modes A_g^1, B_{2g}, and A_g^2 are at 343, 425 and 452 cm^{-1}, respectively. For better visualisation, the intensity of the B_{2g} mode was enhanced by a factor 10^2 in the parallel polarisation configuration.

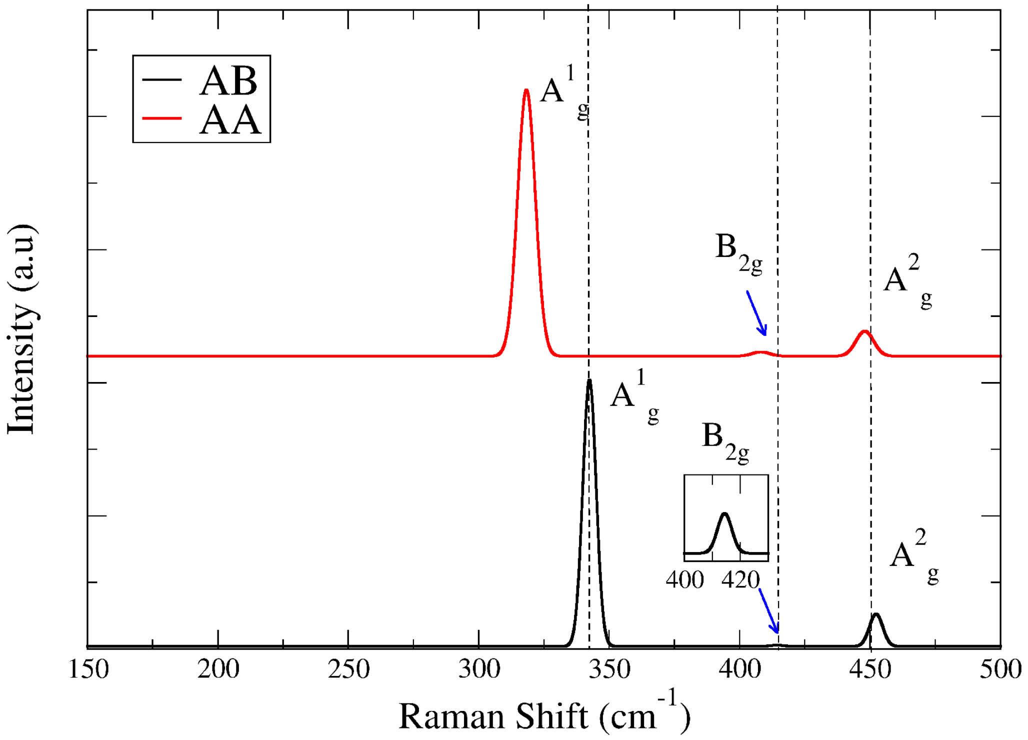

Supplementary Figure 13. **Simulated Raman spectra for AA and AB stacked bulk black phosphorus, for depolarized incident and scattered light.** The vertical dashed lines indicate the position of the corresponding experimental peaks.

SUPPLEMENTARY TABLES

Supplementary Table 1. **Lattice parameters for simulated structures before and after relaxation.** AB-stacking considered in all cases. For the zigzag (armchair) slab the vacuum region in along the a (c) direction. Along that direction the size of the cell does not change (fixed at 30 Å), only the width of the slab inside the cell.

	Initial			Relaxed		
	a (Å)	b (Å)	c (Å)	a (Å)	b (Å)	c (Å)
Bulk	4.55	10.76	3.41	4.55	10.76	3.41
Zigzag slab	-	10.76	3.41	-	10.82	3.31
Armchair slab	4.55	10.76	-	4.33	10.15	-

SUPPLEMENTARY NOTES

Supplementary Note 1 — Additional Experimental Characterisation

Comparison between the XZ and ZX spectra and mode intensity increase at the edges

In this work we performed hyperspectral Raman imaging in black phosphorus single crystal flakes at two different incident polarisations and with two analyser orientations, resulting in four distinct scattering configurations that were labelled in the main manuscript as XX, ZZ, XZ and ZX. Measurements were made on the flake shown in Supplementary Figure **1**(a). As mentioned in the main text, the XZ and ZX configurations present similar trends, and only the former was presented. For completeness, the first and second lines of Supplementary Figure **1**(b) show the hyperspectral images for the XZ and ZX configurations, respectively, for the Raman modes A_g^1, A_g^2, B_{2g}, B_{1g} and B_{3g}^1, as indicated at the top of each column. The Raman intensity discrepancies between the edges and the interior of the flake for all scattering configurations, and for zigzag and armchair edges, can also be observed in Supplementary Figure **1**(c), where the intensity profiles along the solid yellow lines of Supplementary Figure **1**(a) are plotted. These profiles correspond to the intensities averaged along lines parallel to the edges, within the yellow dotted rectangles also shown in Supplementary Figure **1**(a).

Raman spectrum at the bright spots shown in Figure 1(d) of the manuscript

In Supplementary Figure **2** we compare, as an example, the ZZ spectra taken in one of the bright spots in Figure 1(d) of the manuscript and in a perfect region within the interior of the sample. In this configuration only the A_g^1 and A_g^2 are expected to appear. We can observe that the only difference in these two spectra is the presence on an intense background in the bright spot spectrum. As mentioned in the main manuscript text, this background is due to a luminescence process, showing that the bright spots might be related to sample imperfections and/or oxidation[1]. The important point is that the anomalous modes at the edges of the sample are not observed in the bright spots, which indicates that there is no relationship between the spectra at the edges and at the bright spots.

Results obtained in thinner samples

Measurements similar to those presented in Figure 1 of the main manuscript were made in two thinner flakes, with thicknesses of 30 nm and 6 nm. Supplementary Figure **3**(a) shows hyperspectral Raman images for the A_g^1, A_g^2 and B_{2g} modes in the ZZ, XX, XZ and ZX configuration, for the few-layer, 6 nm, flake. In the figure, the zigzag and armchair directions are along the horizontal and vertical axes, respectively. Despite the fact that the Raman signals are much weaker and that the Raman maps are much noisier than those of Fig. 1(d), we can observe the same anomalous results at the edges of the sample. In particular, we note the presence of the B_{2g} mode in the armchair edge for the ZZ configuration, while the A_g modes appear in the armchair edge for the XZ configuration and in the zigzag edge for the ZX configuration. Also, Supplementary Figure **3**(b) shows a comparison between the spectra of the three measured flake thicknesses, for an armchair edge in the XZ configuration. Each spectrum was normalized by its B_{2g} mode intensity. The observation of the A_g modes in the figure is indicative of the change in the atomic structure at the edges, which therefore continues to occur down to few-layer thicknesses. Notice that the amplitude of these modes decreases with crystal thickness. Besides, the non-expected modes, B_{1g} and B_{3g}^1, were not observed for the two thinner flakes since, even for the 300-nm thick flake, their intensities are very low.

Temperature dependence of the edge phonons' Raman response

In order to probe the temperature dependence of the Raman spectra at the edges and away from the edges (at the positions marked in Supplementary Figure **4**(a)), the incident laser power was varied. All phonon frequencies were observed to downshift with increasing laser power. This is an expected behavior for materials with a positive thermal expansion coefficient. Supplementary Figure **4**(b) shows the obtained shifts in A_g^2 mode frequency as a function of the laser power, for the three indicated positions in the sample, while Supplementary Figure **4**(c) shows the shifts for the B_{1g} mode position at the armchair edge. Interestingly, the softening of the A_g^2 phonon frequency with increasing laser power (temperature) is larger at the edges when compared to the interior of the sample, and the curve is steeper for the zigzag edge than for the armchair edge. We interpret these results as a consequence of the anisotropic thermal conductivity in BP [2]. Also, the differences in heat dissipation in the bulk and at edges lead to higher temperatures at the edges than in the sample interior.

Thickness dependence of the relative intensities of the BP and Si Raman peaks

In all measurements, the BP sample was on the top of a Si/SiO_2 substrate, allowing us to observe both the Raman signal of BP and of the Si substrate. Supplementary Figure **11** presents the spectra from flakes with 16 nm (black), 65 nm (blue) and 117 nm (green) thicknesses. We can clearly observe that the Si/BP relative intensities decrease with increasing thickness of the BP sample.

Crystallographic axis determination

It has been recently shown that the determination of the crystallographic axes of BP from the angular dependence of polarized Raman spectroscopy is not necessarily correct, since the Raman tensor elements depend both on the excitation laser energy and on flake thickness [3]. Therefore, the maxima and minima in the angular dependence can be associated with both the armchair or zigzag direction depending on the sample thickness. However, distinction between these two directions can be unambiguously made by the anisotropic optical absorption response of BP (linear dichroism) [4].

In this work, while obtaining the polarized Raman spectra of BP deposited on the top of a silicon substrate (with an oxide top layer), we also analysed the angular intensity dependence of the Si Raman peak at 525 cm^{-1}. First, with an analyser parallel to the polarization of the incident light, the BP crystal was rotated until the amplitude of its B_{2g} Raman band was null. At this orientation the polarization is aligned with either the armchair or zigzag direction [3]. Next, spectra were taken at the flake and away from it (just the substrate). The sample was then rotated by 90° and another pair of spectra was taken. With the excitation wavelength at the visible range, the BP crystal is more absorbing along the armchair direction [5]. Therefore, the ratio between the silicon signal away from and through the flake is higher with polarization parallel to this direction. The solid lines in Supplementary Figure **9** show the Raman spectra through BP, while the dotted curves show the silicon spectrum measured away from the flake. Blue and red curves indicate the two principal orientations of the measured BP flake with a thickness of 170 nm. All spectra were taken using a 488-nm laser line and a 1-mW incident power. It is clear from the figure that, through BP, the silicon mode exhibits lower amplitude in the red curve, which is, thus, assigned to an armchair polarization. The reliability of this method was confirmed through comparison with electron diffraction and HRTEM data, which are presented in the next section

Transmission Electron Microscopy images and diffraction

High-Resolution Transmission Electron Microscopy (HRTEM) images and electron diffraction patterns were obtained in a BP flake directly exfoliated onto a TEM grid, and the results are shown in Supplementary Figure **10**. The HRTEM image of an edge is shown in Supplementary Figure **10**(a). Transmission electron diffraction measurements were performed at the edges and in a region within the interior of the sample and the results are presented in Supplementary Figure **10**(b) and (c), respectively. Notice that, to within experimental resolution, the electron diffraction patterns are the same in these two regions. The lattice parameters evaluated from the diffraction are $a = 0.324nm$ and $b = 0.424nm$ and are in agreement with previous results [6]. The diffraction patterns corroborate the edge determination obtained via our axis determination method.

Supplementary Note 2 — Theory and Methodology

For the theoretical calculations, plane-wave density functional theory was employed to obtain the electronic ground-state using the Perdew-Burke-Ernzerhof (PBE) [7] generalized gradient approximation exchange-correlation functional, currently implemented in the Quantum-Espresso package [8]. Van der Waals corrections within the semi-empirical dispersion scheme (PBE-D) proposed by Grimme [9] were included.

Norm-conserving pseudopotentials with 3s3p states were adopted to describe electronic states of phosphorus. The Brillouin zone was mapped with a k-sampling grid within the Monkhorst-Pack scheme using $7 \times 7 \times 7$ for the bulk and a grid of $10 \times 7 \times 1$ ($1 \times 8 \times 8$) for the armchair (zigzag) surfaces samples. The kinetic energy cutoff was set at 90 Ry and 100 Ry for bulk and slab geometries, respectively. Furthermore a vacuum region of 16 Å was adopted for the supercell related to the surfaces. The structures were fully optimized to their equilibrium position with forces smaller than 0.002 eV Å^{-1} and the unit cells were relaxed to a target pressure of 0.2 kbar. Supplementary Figure **5** shows the structures obtained after relaxation highlighting the unit cell used in the calculations. Supplementary Table **1** presents the unit cell lattice parameters before and after relaxation for bulk and for both edge terminations.

Within the linear response framework, the lattice distortion induced by phonons can be considered as a static perturbation acting upon the electrons. This enables the construction of the matrix elements for harmonic interatomic force constants

$$C_{\alpha i,\beta j}(\mathbf{R}-\mathbf{R}^{'}) = \frac{\partial^2 \mathcal{E}}{\partial u_{\alpha i}(\mathbf{R}) \partial u_{\beta j}(\mathbf{R}^{'})}, \tag{1}$$

where $\mathcal{E}$ is the electronic ground-state energy and $u_{\alpha i}(\mathbf{R})$ represent the ith atom displacement in the αth direction at position $\mathbf{R}$ of the unit cell. By performing the Fourier transform of equation 1 one is able to solve the secular equation

$$\sum_{\beta j} w_{\beta j}(\mathbf{q}) D_{\alpha i,\beta j}(\mathbf{q}) = \omega^2(\mathbf{q}) w_{\alpha i}(\mathbf{q}), \tag{2}$$

to obtain the phonon frequencies ω and normal modes $w_{\alpha i}$. Here the dynamical matrix $D_{\alpha i,\beta j}(\mathbf{q})$ stands for the Fourier transform of the interatomic force constants.

The linear response approach can also be used to calculate the dielectric tensor (response

function)

$$\epsilon^{\infty}_{lm} = \delta_{lm} - \frac{4\pi}{\Omega}\frac{\partial^2 \mathcal{E}^{\mathrm{el}}}{\partial E_l \partial E_m}, \quad (3)$$

which relates the bare electric field $\mathbf{E}_0$ with the screened one $\mathbf{E}$. In equation 3, $\mathcal{E}^{\mathrm{el}}$ is the electronic energy of the system in the presence of a uniform electric field $E_{l(m)}$ along direction $l(m)$, and Ω is the system volume. As we will see bellow, the dielectric tensor is a crucial observable in determining the Raman intensities.

Within the Placzek approximation [10], the Raman intensity of a particular normal mode ν is calculated as:

$$I^{\nu} \propto |\mathbf{e}_{\mathrm{i}} \ . \ \mathbf{A}^{\nu} \ . \ \mathbf{e}_{\mathrm{s}}|^2 \frac{1}{\omega_\nu}(n_\nu + 1) \ , \quad (4)$$

where $\mathbf{e}_{\mathrm{i}}$ ($\mathbf{e}_{\mathrm{s}}$) is the incident (scattered) light polarization, n_ν is the Bose-Einstein distribution, and $\mathbf{A}^{\nu}$ is the Raman tensor with matrix elements:

$$A^{\nu}_{lm} = \sum_{k\gamma} \frac{\partial^3 \mathcal{E}^{\mathrm{el}}}{\partial E_l \partial E_m \partial u_{k\gamma}} \frac{w^{\nu}_{k\gamma}}{\sqrt{M_\gamma}} \ . \quad (5)$$

In equation 5, M_γ corresponds to the atomic mass of the $\gamma - th$ atom, $\mathcal{E}^{\mathrm{el}}$ is the electronic energy of the system, E_l is the component of the electric field along direction l, $u_{k\gamma}$ is the displacement of the γth atom in the kth direction and $w^{\nu}_{k\gamma}$ is the orthonormal vibrational eigenmode ν [10]. Further details of the methodology are given in Ref [11].

The Raman tensor for all the structures were obtained using the PHonon code, currently integrated into the Quantum-Espresso package. It relies on the perturbative treating of electron-phonon interaction, in what is known as density functional perturbation theory (DFPT) [12].

For a quantitative analysis of the Raman intensities dependency on polarisation, we defined the polarisation vector for the incident light as $\mathbf{e}_i = (sin\theta \ \ 0 \ \ cos\theta)^T$, the vector for scattered light in the same polarisation as $\mathbf{e}_{\mathrm{s}}^{\parallel} = (sin\theta \ \ 0 \ \ cos\theta)^{\mathrm{T}}$ and the vector for scattered light at the orthogonal polarisation as $\mathbf{e}_{\mathrm{s}}^{\perp} = (-cos\theta \ \ 0 \ \ sin\theta)^{\mathrm{T}}$ (the angle θ is measured with respect to z crystalographic axis, i.e., the zigzag direction).

Considering a generalized Raman tensor

$$\mathbf{A} = \begin{pmatrix} a & b & c \\ b & n & p \\ c & p & f \end{pmatrix} \quad (6)$$

one can obtain the following expressions for the measurable intensities as a function of the angle θ:

$$I^{\parallel} = |\mathbf{e}_{\mathrm{i}} \ . \ \mathbf{A} \ .\mathbf{e}_{\mathrm{s}}^{\parallel}|^2 = [a\sin\theta^2 + f\cos\theta^2 + 2c\sin\theta\cos\theta]^2 \tag{7}$$

$$I^{\perp} = |\mathbf{e}_{\mathrm{i}} \ . \ \mathbf{A} \ .\mathbf{e}_{\mathrm{s}}^{\perp}|^2 = [c(\sin\theta^2 - \cos\theta^2) + (f-a)\sin\theta\cos\theta]^2 \tag{8}$$

Bulk black phosphorus

In Supplementary Figure **6**, we show the crystalline structure of AB-stacked black phosphorus as well as the atomic displacements of the six active Raman modes obtained within the DFPT formalism. The calculated Raman frequencies for the A_g^1, B_{2g}, and A_g^2 modes are 343, 425 and 452 cm^{-1}, respectively. We also performed calculations using the AA stacking. Our simulations, as well as others' [13], show that the AB stacking is a few meV per atom more stable. Furthermore, Supplementary Figure **13** shows the Raman spectra simulated using DFPT for both stackings compared to our experiments. There we can see that the AB-stacked structure yields the best agreement with the experimental results (dashed vertical lines).

The angular dependence of the Raman spectra can be seen in Supplementary Figures **12**(a) and **12**(b) for the parallel and crossed polarisation configurations, respectively; it is evident that only modes A_g^1, B_{2g}, and A_g^2 are observable, as predicted by group theory, because incidence is assumed to be along the y axis, as in the experiments.

Edge structures

For studying the edges of black phosphorus, we first calculated the atomic vibrational modes for both zigzag and armchair slabs by solving equation 2, and compared their intensities with those for bulk. We then looked for edge enhancements similar to those that were experimentally observed. Even though simulation-experiment agreement was not achieved for all modes in all scattering configurations, several experimental observations were qualitatively reproduced. Here, we highlight the situations for which such an agreement is achieved. We begin our analysis by considering the zigzag case. There are 72 vibrational modes, which

include confined, inactive, and active modes. We identify the Raman modes observed in the experiment by adopting two criteria: (i) we focus on a range of frequencies close to those calculated for the Raman active modes of the bulk material and (ii) we compare the atomic displacement vectors of the slab with those found for the bulk case. The results were presented and discussed in Figure 3 of the main manuscript.

In Supplementary Figure **7**, we compare the Raman intensities of the bulk with those obtained simulating the zigzag slab, for the ZZ, XX, and XZ scattering configurations. The totally symmetric Raman modes for the slab arise at A_g^1 = 344.6 cm^{-1} and A_g^2 = 441.7 cm^{-1}, whereas the non-totally symmetric modes B_{1g}, B_{3g}^1, B_{2g} emerge at 175.4, 224.6 and 417 cm^{-1} respectively. Notice that the frequency related to the Aby 10 cm^{-1} with respect to the bulk, which might be associated to the quantum confinement induced by the formation of the slab. For the ZZ configuration obtained theoretically, we observed that the non-totally symmetric modes did not present any significant enhancement associated to the zigzag edge since their intensities are up to 3 orders of magnitude smaller than those of the totally symmetric modes. In contrast, mode A_g^1 shows a clear Raman intensity enhancement due to the presence of the edge. A close view of mode A_g^2 also shows a slight enhancement at the edge. For the XX configuration, there are no significant zigzag edge effects associated to the A_g^2 mode. On the other hand, we clearly observed an edge enhancement for modes B_{3g}^1 and B_{2g}. Finally, the XZ configuration shows that modes B_{1g} and B_{2g} possess the dominant intensities. In particular, one can clearly see a robust enhancement of the B_{1g} intensity at the edge. In addition, the totally-symmetric modes present weaker, albeit noticeable, enhancement effects at the zigzag edge.

In Supplementary Figure **8** we show the Raman intensities for the armchair edge with the ZZ, XX, and ZX configurations. For the ZZ configuration a noticeable intensity enhancement is observed for the totally-symmetric mode A_g^2 and for the non-totally symmetric modes B_{1g} and B_{2g}. In addition, for configuration ZX the most enhanced mode is B_{3g}^1 whereas mode B_{2g} does not show significant enhancement due to the edge.

SUPLEMENTARY REFERENCES

---

[1] Lu, J. *et al.* Bandgap engineering of phosphorene by laser oxidation toward functional 2d materials. *ACS Nano* **9**, 10411–10421 (2015).

[2] Lee, S. *et al.* Anisotropic in-plane thermal conductivity of black phosphorus nanoribbons at temperatures higher than 100 K. *Nature Communications* **6**, 8573 (2015).

[3] Kim, J. *et al.* Anomalous polarization dependence of Raman scattering and crystallographic orientation of black phosphorus. *Nanoscale* **7**, 18708–18715 (2015).

[4] Ling, X. *et al.* Anisotropic electron-photon and electron-phonon interactions in black phosphorus. *Nano Letters* **16**, 2260–2267 (2016).

[5] Mao, N. *et al.* Optical anisotropy of black phosphorus in the visible regime. *Journal of the American Chemical Society* **138**, 300–305 (2016).

[6] Luo, Z. *et al.* Anisotropic in-plane thermal conductivity observed in few-layer black phosphorus. *Nature Communications* **6**, 8572 (2015).

[7] Perdew, J. P., Burke, K. & Ernzerhof, M. Generalized gradient approximation made simple. *Phys. Rev. Lett.* **77**, 3865–3868 (1996).

[8] Giannozzi, P. & et al. Quantum espresso: a modular and open-source software project for quantum simulations of materials. *J. Phys. Condens. Matter* **21**, 395502 (2009).

[9] Grimme, S. Semiempirical GGA-*type* density functional constructed with a long-range dispersion correction. *J. Comput. Chem.* **27**, 1787–1799 (2006).

[10] Lazzeri, M. & Mauri, F. First-principles calculation of vibrational raman spectra in large systems: Signature of small rings in crystalline SiO_2. *Phys. Rev. Lett.* **90**, 036401 (2003).

[11] Porezag, D. & Pederson, M. R. Infrared intensities and Raman-scattering activities within density-functional theory. *Phys. Rev. B* **54**, 7830–7836 (1996).

[12] Baroni, S., de Gironcoli, S., Dal Corso, A. & Giannozzi, P. Phonons and related crystal properties from density-functional perturbation theory. *Rev. Mod. Phys.* **73**, 515–562 (2001).

[13] Dai, J. & Zeng, X. C. Bilayer phosphorene: Effect of stacking order on bandgap and its potential applications in thin-film solar cells. *J. Phys. Chem. Lett.* **5**, 1289–1293 (2014).
